# Impact of the Spanish Smoking Law on Exposure to Second-Hand Smoke and Respiratory Health in Hospitality Workers: A Cohort Study

**DOI:** 10.1371/journal.pone.0004244

**Published:** 2009-01-23

**Authors:** Esteve Fernández, Marcela Fu, José A. Pascual, María J. López, Mónica Pérez-Ríos, Anna Schiaffino, Jose M. Martínez-Sánchez, Carles Ariza, Esteve Saltó, Manel Nebot, and the Spanish Smoking Law Evaluation Group

**Affiliations:** 1 Tobacco Research & Control Unit, Institut Català d'Oncologia (ICO), L'Hospitalet de Llobregat, Barcelona, Spain; 2 Department of Clinical Sciences, Campus of Bellvitge, Universitat de Barcelona, L'Hospitalet, Barcelona, Spain; 3 Institut d'Investigació Biomèdica de Bellvitge (IDIBELL), L'Hospitalet de Llobregat, Barcelona, Spain; 4 Department of Experimental and Health Sciences, Universitat Pompeu Fabra, Barcelona, Spain; 5 Bioanalysis Research Group, Neuropsychopharmacology Programme, IMIM-Hospital del Mar, Barcelona, Spain; 6 Evaluation and Intervention Methods Unit, Agència de Salut Pública de Barcelona (ASPB), Barcelona, Spain; 7 CIBER de Epidemiología y Salud Pública (CIBERESP), Barcelona, Spain; 8 Programme in Public Health and Biomedical Research, Universitat Autònoma de Barcelona, Bellaterra, Spain; 9 Consejería de Salud, Xunta de Galicia, Santiago de Compostela, Spain; 10 Department of Public Health, Universidade de Santiago de Compostela, Santiago de Compostela, Spain; 11 Ajuntament de Terrassa-IMSABS, Terrassa, Spain; 12 Department of Public Health, Universitat de Barcelona, Barcelona, Spain; 13 Department of Health, Generalitat de Catalunya, Barcelona, Spain; National Institute for Public Health and the Environment, Netherlands

## Abstract

**Background:**

A smoke-free law came into effect in Spain on 1st January 2006, affecting all enclosed workplaces except hospitality venues, whose proprietors can choose among totally a smoke-free policy, a partial restriction with designated smoking areas, or no restriction on smoking on the premises. We aimed to evaluate the impact of the law among hospitality workers by assessing second-hand smoke (SHS) exposure and the frequency of respiratory symptoms before and one year after the ban.

**Methods and Finding:**

We formed a baseline cohort of 431 hospitality workers in Spain and 45 workers in Portugal and Andorra. Of them, 318 (66.8%) were successfully followed up 12 months after the ban, and 137 nonsmokers were included in this analysis. We obtained self-reported exposure to SHS and the presence of respiratory symptoms, and collected saliva samples for cotinine measurement. Salivary cotinine decreased by 55.6% after the ban among nonsmoker workers in venues where smoking was totally prohibited (from median of 1.6 ng/ml before to 0.5 ng/ml, p<0.01). Cotinine concentration decreased by 27.6% (p = 0.068) among workers in venues with designated smoking areas, and by 10.7% (p = 0.475) among workers in venues where smoking was allowed. In Portugal and Andorra, no differences between cotinine concentration were found before (1.2 ng/ml) and after the ban (1.2 ng/ml). In Spain, reported respiratory symptom declined significantly (by 71.9%; p<0.05) among workers in venues that became smoke-free. After adjustment for potential confounders, salivary cotinine and respiratory symptoms decreased significantly among workers in Spanish hospitality venues where smoking was totally banned.

**Conclusions:**

Among nonsmoker hospitality workers in bars and restaurants where smoking was allowed, exposure to SHS after the ban remained similar to pre-law levels. The partial restrictions on smoking in Spanish hospitality venues do not sufficiently protect hospitality workers against SHS or its consequences for respiratory health.

## Introduction

Several countries have limited the hazards of second-hand smoke (SHS) for health with legislation intended to ensure smoke-free workplaces in enclosed public places [1], [2]. Smoke-free workplaces not only protect nonsmokers from SHS, but may stimulate smokers to quit or smoke less [3]. Prompted by compelling evidence, the government of Spain introduced a comprehensive ban on smoking in public places on January 1st, 2006 (Law 28/2005) [4], [5]. The law is a compendium of public health measures against smoking and includes regulations on the advertising, sale, supply, and consumption of tobacco products. Smoking is now banned in all indoor workplaces, public places, public transport facilities including enclosed stations, hospitals and other health care facilities, schools and universities, as well as in retail stores and shopping centres. However, hospitality venues are subject to only a partial ban [6]. Bars and restaurants larger than 100 m^2^ are defined as smoke-free, but the law allows the proprietor to provide a physically separate and independently ventilated smoking area comprising less than 30% of the total floor area. For “small” venues with a floor area below 100 m^2^ the owner may choose whether to be smoke-free or not, and it is estimated that only 10%–20% of such venues have banned smoking [7].

Evaluations of the impact of total bans on smoking in other countries have shown clear reductions in SHS exposure and improvements in the respiratory health of hospitality workers [8]–[18]. In Spain, however, these potential benefits await confirmation, given that the partial ban creates a “natural experiment” in which a large proportion of hospitality workers continue to work in venues where smoking is allowed, while others now work in completely smoke-free environments. The importance of determining the real impact of the Spanish law on hospitality workers goes beyond Spanish borders, since other countries have adopted or are considering similar partial bans instead of total bansn [19]–[24].

We evaluated biologically assessed and self-reported exposure to SHS and respiratory health in hospitality workers in five regions of Spain before and after the law came into effect. As a control group we studied hospitality workers in Portugal and Andorra, where no ban on smoking was in effect at that time.

## Methods

We included hospitality workers (employed at pubs, bars, restaurants, hotels and discotheques) in Spain, Portugal and Andorra, in a baseline survey during the three months before the law came into effect (October–December 2005) [25] and followed them up 6 months (April–June 2006) and 12 months later (October–December 2006). We assessed changes in exposure to SHS and respiratory symptoms according to the type of regulation in Spanish venues after the law (smoking completely prohibited, permitted in restricted areas, or permitted in the entire venue), and in Portugal and Andorra as control areas.

### Participant recruitment and sample size

The study took place in five regions within Spain (Balearic Islands, Cantabria, Catalonia, Galicia, and Valencia), and in Portugal (city of Braga) and Andorra (municipalities of Andorra la Vella and Encamp). We selected Portugal and Andorra as control areas since they had no ban on smoking at the time of the study, and because no control sample of hospitality venues was available in Spain [26]–[29].

The study included 4 different types of venue: pubs, bars, restaurants and discos. For each type we used a nonproportional quota sampling method based on their size (smaller or larger than 100 m^2^) and area (urban, rural, tourist and nontourist). The specific premises for each type of venue were selected for convenience, based on their accessibility to the researchers and whether the owners agreed to participate. If a venue refused to participate in the study, it was substituted by another one with similar characteristics. We contacted 342 venues, and 215 (62.9%) participated in the study. We used this sampling approach because of difficulties with access comprehensive censuses, and because of the need to maximize recruitment for the study. To be eligible, participants had to work at least 6 hours per day, had to be employed at the same venue for more than one year before the baseline survey, and had to report no intention of changing jobs in the subsequent two years. After we obtained the proprietor's permission, up to 6 workers (managers, owners and staff) present at the time of the initial site visit were invited to participate. An equal number of smokers and nonsmokers from each venue were recruited for the final sample.

Although we enrolled both smokers and nonsmokers, the present analysis is restricted to workers who were nonsmokers (never or former smokers) at both baseline and follow-up. The presented analysis refers to the 12 month follow-up, since the 6 month follow-up (April–June 2006) might have been influenced by seasonality and by the transition period allowed by the law to adapt venues (physical isolation of smoking areas was not compulsory before September 1st 2006).

To achieve a sufficient sample size in the light of potential attrition as observed in previous studies, we estimated the sample size as 480 workers (440 in Spain and 40 in Portugal and Andorra). After the baseline survey, the cohort of hospitality workers consisted of 431 workers in Spain (202 nonsmokers and 229 smokers) and 45 workers in Portugal and Andorra (32 nonsmokers and 13 smokers) at 215 venues.

### Field work

We contacted the venues' owner or manager, and after obtaining their permission, contacted the workers during their work shift. We briefly explained the overall aim of the study and the type of assistance we were requesting, provided a letter of presentation, and obtained written informed consent before proceeding with the survey and saliva collection. Baseline and follow-up face-to-face interviews and saliva samples were obtained at the workplace across a range of weekday and weekend days (with up to 5 attempts for follow-up).

### Ethics statement

The research and ethics committee of the Bellvitge University Hospital provided ethical approval for the study protocol, including the informed consent form.

### Exposure to second-hand smoke and active smoking

#### Salivary cotinine

We obtained a saliva sample according to a previously described protocol [30], [31]. Participants were asked to rinse their mouth and then suck a lemon-flavored candy (Smint^®^) to stimulate saliva production. They were asked to spit out a small amount of saliva and then to provide about 8 ml by spitting into a funnel placed in a test tube. The tubes were kept at 4°C until they were sent by express courier to the coordinating center (Catalan Institute of Oncology) in Barcelona, where saliva was separated in 3-ml aliquots and frozen at −20°C for storage. The frozen samples were sent to the Bioanalysis Research Group of the Municipal Institute for Medical Research (IMIM-Hospital del Mar) in Barcelona, where salivary cotinine was measured by capillary gas chromatography and mass spectrometry [32], [33] with a limit of quantification of 1 ng/ml.

#### Smoking and perceived exposure to second-hand smoke

We collected information on smoking status, number of cigarettes smoked per day, and type of cigarettes smoked. We asked about exposure to SHS at work, at home, and during leisure time during the 7 days before the interview, and recorded responses separately for working and nonworking days [34]–[37] (see Annex S1). For the purposes of analysis we re-coded the information into two variables: exposure to SHS at work and exposure to SHS in other places (in hours/day). We also recorded the day of the week of the interview (and date), the number of hours worked per day, and the type of smoking regulation in effect at venue (total ban, partial restriction, no restriction).

### Respiratory symptoms

We used the *European Community Respiratory Health Study (ECRHS)* questionnaire [38], [39] to assess respiratory health, and considered the eight main symptoms (recall period last 12 months): breathless while wheezing, woken up with a feeling of chest tightness, attack of shortness of breath at rest, woken by attack of shortness of breath, usually cough first thing in the morning in winter, usually cough during the day or night during winter, usually bring up phlegm during day or night in winter, had asthma attack. We computed the prevalence of each symptom individually and combined all symptoms into a single indicator variable (presence/absence of any of the eight respiratory symptoms).

### Statistical analysis

We defined as nonsmokers those workers who said they were nonsmokers (never or former smokers) at the time of the interview and who had salivary cotinine concentrations <20 ng/ml, since exposure to SHS in hospitality workers can be high [40] as shown in previous research [10]. Given the paired nature of the data (pre-post comparisons), analyses were restricted to participants with complete information at baseline and 12-month follow-up, who continued to work at hospitality venues, and were nonsmokers at both baseline and follow-up. We used median and interquartile ranges given that the distribution of salivary cotinine concentration was highly skewed to the right. For unpaired comparisons we used Wilcoxon's rank sum test and Fisher's exact test. For paired comparisons we used Wilcoxon's signed rank test to compare medians before and after the ban, and McNemar's chi-squared test to compare the frequency of symptoms before and after the ban. To report the magnitude of the changes observed, we calculated average percentages of change (before-after) and 95% confidence intervals (CI) for salivary cotinine concentrations and frequency of respiratory symptoms from simple linear and logistic regression models. Univariate and bivariate analyses were conducted with SPSS v.13 (SPSS Inc., Chicago, IL).

Changes in salivary cotinine concentration and frequency of symptoms may be confounded by time-independent variables (i.e., sex, geographical area or workers' clustering within venues) as well as by time-dependent variables (body mass index, self-reported exposure to SHS at work and other places, number of hours worked per day, day and month of the interview). We used generalized least squares regression models with random effects to model the changes in salivary cotinine concentrations (after log10 transformation) and control for time-dependent and -independent confounders and self-correlation between before and after measurements [41], [42]. Adjusted percentages of change (and 95% CI) were calculated for salivary cotinine from the model coefficients. We used logistic regression models with random effects to model changes in the prevalence of respiratory symptoms and control for time-dependent and -independent confounders and self-correlation between before and after measurements. Adjusted percentages of change (and 95% CI) were calculated for the prevalence of any respiratory symptom from the model coefficients, taking into account baseline prevalence rates (since the logistic regression model overestimates associations when the prevalence of exposure is >20%) [43]. Multivariate analyses were performed with Stata 9 software (StataCorp, College Station, TX).

## Results

We recruited 476 hospitality workers (431 in Spain and 45 in Portugal and Andorra) in the baseline survey, and 318 (288 in Spain and 30 in Portugal and Andorra) were followed up 12 months later (overall follow-up rate of 66.8%). In Spain, 143 workers were lost to follow-up: 70 were not located after 5 attempts, 31 declined to participate in follow-up, 33 changed jobs, 6 were on sick leave and 3 were unemployed (follow-up rate of 66.8%). In Portugal and Andorra, 15 workers were lost to follow-up: 8 were not located after 5 attempts, 2 declined to participate in follow-up, 4 changed jobs and 1 was on sick leave (follow-up rate of 66.7%). Workers lost to follow-up in Spain were younger, more frequently of foreign origin, and more frequently smokers, whereas in the control areas there were no differences (data not shown). For the present analysis we excluded smokers (133 in Spain and 10 in Portugal and Andorra), defined as participants who identified themselves as smokers at baseline or follow-up, or who had a salivary cotinine concentration ≥20 ng/ml. In Spain we excluded 38 nonsmokers who were already working in smoke-free venues before the anti-smoking law came into effect. The final cohort consisted of 137 nonsmoking workers (117 in Spain and 20 in Portugal and Andorra) whose baseline characteristics are shown in Table 1. Median salivary concentration was greater in Spain than in Portugal or Andorra (2.0 ng/ml vs. 1.2 ng/ml, p<0.01). In Spain, pre-law salivary cotinine concentrations were lower in workers whose venues became totally or partly smoke-free after the law took effect, compared to those who worked in venues where smoking was still allowed after 1st January 2006 (1.65 ng/ml vs. 2.50 ng/ml; p<0.05).

**Table 1:** Baseline characteristics of nonsmoker* hospitality workers who completed follow-up at 12 months.

	Spain	Portugal and Andorra	p-value†
	n = 117	n = 20	
Age, median (IQR) (years)	39.4 (30.9–48.8)	37.1 (31.6–41.8)	0.373
Sex, n (%) of women	46 (39.3)	14 (70.0)	0.014
Hours/day worked, median (IQR)	9.0 (8.0–10.0)	9.0 (8.0–12.0)	0.718
Salivary cotinine concentration (ng/ml), median (IQR)	2.0 (1.4–3.1)	1.2 (0.6–1.6)	<0.01
Self-reported exposure to second-hand smoke, hours/day:			
At work, median (IQR)	8.0 (0.0–10.0)	8.0 (4.5–8.0)	0.797
Outside work, median (IQR)	0.5 (0.0–1.5)	0.1 (0.0–0.5)	0.043
Prevalence of any respiratory symptom, n (%) ‡	38 (32.5)	14 (70.0)	<0.01

* Defined as workers who reported they were former or never smokers in the baseline and follow-up interviews, and had salivary cotinine concentrations <20 ng/ml. (Workers who changed smoking status between surveys were considered smokers and hence excluded from this analysis.)

† p-values for comparison of medians (Wilcoxon's test for independent samples) and categorical variables (Fisher's exact test).

‡ Any of the following: breathless while wheezing, woken up with a feeling of chest tightness, attack of shortness of breath at rest, woken by attack of shortness of breath, usually cough first thing in the morning in winter, usually cough during the day or night during winter, usually bring up phlegm during day or night in winter, had asthma attack.

IQR: interquartile range

In the Spanish cohort, salivary cotinine concentration decreased significantly (by 56.6%) among workers at venues where smoking was totally banned after the law took effect, from median of 1.6 ng/ml to 0.5 ng/ml (p<0.01; Table 2). Cotinine decreased nonsignificantly by 31.9% and 1.6%, respectively, in venues with designated smoking areas and in venues without smoking restrictions. In Portugal and Andorra, no changes in median salivary cotinine concentration were seen after the law came into effect.

**Table 2:** Exposure to second-hand smoke in nonsmoker* hospitality workers reported in baseline and follow-up surveys in Spain and Portugal & Andorra.

	n	Baseline†	Follow-up†	p-value‡	% change (95% confidence interval)¶
**SPAIN (according to type of post-ban regulation)** **					
Salivary cotinine, ng/ml					
Smoking completely banned	32	1.6 (1.2–2.2)	0.5 (0.5–1.1)	<0.01	−56.6 (−63.7;−48.0)
Smoking permitted in designated areas	22	1.8 (1.2–3.0)	1.1 (0.8–1.7)	0.068	−31.9 (−53.7;0.3)
Smoking permitted throughout the premises	63	2.5 (1.7–3.9)	2.6 (1.7–3.7)	0.475	−1.6 (−15.2;14.2)
Self-reported exposure to second-hand smoke at work, hours/day					
Smoking completely banned	30	3.0 (0.0–8.0)	0.0 (0.0–0.0)	<0.01	−100.0 (––)
Smoking permitted in designated areas	19	8.0 (0.0–9.0)	1.0 (0.0–8.0)	0.055	−47.8 (−71.1;−6.0)
Smoking permitted throughout the premises	58	8.0 (0.7–10.0)	10.0 (8.0–12.0)	<0.01	10.2 (−4.3;26.8)
Self-reported exposure to second-hand smoke in other settings, hours/day					
Smoking completely banned	30	0.5 (0.0–1.5)	0.5 (0.0–0.9)	0.013	−35.7 (−55.8;−6.6)
Smoking permitted in designated areas	19	0.5 (0.1–1.5)	0.0 (0.0–0.1)	<0.01	−54.6 (−80.1;−3.7)
Smoking permitted throughout the premises	59	0.5 (0.0–1.7)	0.3 (0.0–0.9)	0.061	−16.1 (−38.9;−3.8)
**PORTUGAL & ANDORRA (control areas)**					
Salivary cotinine, ng/ml	20	1.2 (0.6–1.6)	1.2 (0.5–1.6)	0.962	−9.5 (−33.9; 23.9)
Self-reported exposure to second-hand smoke at work, hours/day	19	8.0 (4.5–8.0)	8.0 (8.0–9.5)	0.180	18.2 (−2.3; 43.0)
Self-reported exposure to second-hand smoke in other settings, hours/day	19	0.04 (0.0–0.6)	0.1 (0.0–1.0)	0.463	54.2 (6.2; 123.9)

* Defined as workers who reported they were former or never smokers in the baseline and follow-up interviews, and had salivary cotinine concentrations <20 ng/ml. (Workers who changed smoking status between surveys were considered smokers and hence excluded from this analysis.)

** In Spain, smoking was permitted in all venues at baseline (before the law entered into effect), and was completely banned, permitted in designated areas, or permitted throughout the premises at follow-up. In Portugal and Andorra, smoking was allowed without restrictions at both baseline and follow-up.

† Values are medians (interquartile ranges).

‡ p-values for comparison of medians (Wilcoxon's test for paired samples) and categorical variables (McNemar's chi-squared test).

¶ Percentage change derived from a simple linear regression model with random effects.

Self-reported exposure to SHS at work showed the greatest decrease (100%) in Spanish venues were smoking was totally banned, whereas a borderline-significant decrease (from 8 hours of median exposure per day before the law to 1 hour per day) occurred in venues where smoking was partially permitted after the law. Median exposure increased significantly in venues with no smoking restrictions (Table 2). In Portugal and Andorra, SHS exposure at work did not change after the law came into effect. Second-hand smoke exposure outside the workplace decreased in Spain regardless of the type of post-ban regulation, whereas it increased in Portugal and Andorra (Table 2).

The baseline prevalence of each symptom considered individually (breathless while wheezing, 7.4%; woken up with a feeling of chest tightness, 11.0%; attack of shortness of breath at rest, 8.0%; woken by attack of shortness of breath, 6.1%; asthma attack, 3.7%) did not significantly change after the ban in Spain regardless of the type of post-ban smoking regulation, except for cough and phlegm among workers in totally smoke-free venues (from 40.6% to15.6% considered together, p<0.05). No changes were observed in the control regions for individual symptoms (data not shown). The prevalence of any respiratory symptom before the law was 32.5% (95% CI 24.0–41.0%) in Spain. After the law came into effect, this pre-ban prevalence differed depending on the type of restriction (Table 3). Among workers in completely smoke-free venues, self-reports of any respiratory symptom in Spain declined significantly, but not in workers in venues where smoking was allowed on part or all of the premises (Table 3). In Portugal and Andorra, a borderline-significant decrease was observed.

**Table 3:** Presence of respiratory symptoms in nonsmoker* hospitality workers at baseline and follow-up in Spain and Portugal & Andorra.

	n	Baseline†	Follow-up†	p-value‡	% change (95% confidence interval)¶
**SPAIN (according to type of post-ban regulation)** **					
Presence of any respiratory symptom, n (%)§					
Smoking completely banned	32	56.3 (39.1–73.4)	28.1 (12.5–43.7)	0.012	−71.9 (−94.6; −13.2)
Smoking permitted in designated areas	22	18.2 (2.1–34.3)	9.1 (0.0–21.1)	0.625	−57.1 (−94.7; 74.8)
Smoking permitted throughout the premises	63	25.4 (14.6–36.1)	22.2 (12.0–32.5)	0.774	−19.4 (−67.1; 51.5)
**PORTUGAL & ANDORRA (control areas)**					
Presence of any respiratory symptom, n (%)§	20	70.0 (49.9–90.1)	40.0 (18.5–61.5)	0.070	−61.9 (−95.2; −0.2)

* Defined as workers who reported they were former or never smokers in the baseline and follow-up interviews, and had salivary cotinine concentrations <20 ng/ml. (Workers who changed smoking status between surveys were considered smokers and hence excluded from this analysis.)

** In Spain, smoking was permitted in all venues at baseline (before the law entered into effect), and was completely banned, permitted in designated areas, or permitted throughout the premises at follow-up. In Portugal and Andorra, smoking was allowed without restrictions at both baseline and follow-up.

† Values are percentages and 95% confidence intervals.

‡ p values for comparison of categorical variables (McNemar's chi-squared test).

¶ Percentage change derived from a logistic regression model with random effects. Percentage change corrected for baseline prevalence of any symptom.

§ Any of the following: breathless while wheezing, woken up with a feeling of chest tightness, attack of shortness of breath at rest, woken by attack of shortness of breath, usually cough first thing in the morning in winter, usually cough during the day or night during winter, usually bring up phlegm during day or night in winter, had asthma attack.

After adjustment for potential confounders (Table 4), salivary cotinine concentration decreased significantly by 63.7% after the ban among workers in venues where smoking was completely prohibited in Spain, whereas nonsignificant changes were found among workers in venues where smoking was permitted on part (20.3% decrease) or all of the premises (20.6% increase). To further study this effect, we fitted a model for all the workers including, in addition to the rest of covariates, an indicator variable for the type of regulation after the ban. Hence, relative to those workers in totally smoke-free venues after the ban, there was a nonsignificant increase of 3.1% (95% CI −24.5–40.7%) in salivary cotinine concentrations among workers in venues with smoking areas, and a significant increase of 80.9% (95% CI 37.4–140.3%) in workers in venues without smoking restrictions.

**Table 4:** Multivariate models for the changes in salivary cotinine concentrations and prevalence of any respiratory symptom between baseline and follow-up in Spain and Portugal & Andorra.

	Regression coefficient (standard error)	p-value	Adjusted % change (95% confidence interval)
**SPAIN (according to type of post-ban regulation)** *			
Salivary cotinine concentration **			
Smoking completely banned	−0.439 (0.045)	<0.001	−63.7(−70.4; −55.3)
Smoking permitted in designated areas	−0.098 (0.118)	0.406	−20.3 (−53.3; 36.0)
Smoking permitted throughout the premises	0.081 (0.053)	0.126	20.6 (−5.1; 53.2)
Presence of any respiratory symptom, n (%)†			
Smoking completely banned	−4.784 (1.624)	<0.001	−98.1 (−99.9; −51.3)
Smoking permitted in designated areas	−2.229 (1.509)	0.140	−78.4 (−99.1; 15.9)
Smoking permitted throughout the premises	−0.278 (0.595)	0.640	−19.3 (−70.7; 60.3)
**PORTUGAL & ANDORRA (control areas)** *			
Salivary cotinine concentration **	0.014 (0.174)	0.937	3.2 (−52.9; 126.4)
Presence of any respiratory symptom, n (%)†	−0.639 (2.065)	0.757	−28.1 (−98.5; 34.6)

* In Spain, smoking was permitted in all venues at baseline (before the law entered into effect), and was completely banned, permitted in designated areas, or permitted throughout the premises at follow-up. In Portugal and Andorra, smoking was allowed without restrictions at both baseline and follow-up.

** Adjusted for sex, age, body mass index, self-reported second-hand smoke exposure at work and other settings, number of hours worked, geographical area, day and moth of saliva collection by generalized least squared regression with random effects.

† Adjusted for sex, age, body mass index, self-reported second-hand smoke exposure at settings other than the workplace, number of hours worked, and geographical area by logistic regression with random effects. Percentage change corrected for baseline prevalence of any symptom.

Multivariate adjustment confirmed the lack of significant changes between baseline and follow-up salivary cotinine concentrations in Portugal and Andorra. The presence of any respiratory symptom significantly decreased in hospitality workers in venues where smoking was completely prohibited in Spain, whereas no significant changes were found in the rest of workers in Spain, or in Portugal and Andorra (Table 4).

## Discussion

This study shows that the Spanish anti-smoking law had variable effects in workers at hospitality venues. At venues where smoking was completely prohibited we found a significant reduction in salivary cotinine concentration, in self-reported exposure to SHS, and in respiratory symptoms, whereas no changes were seen in workers at venues where smoking was only partially restricted or permitted throughout the premises. In addition, no reductions were observed in workers in Portugal and Andorra, where no ban was in force at the time of this study. Although the Spanish law was conceived to protect the workers' health [4], our results suggest that exceptions to the ban in the hospitality sector (proprietors can chose to permit smoking in small venues and allow smoking in designated areas in venues larger than 100 m^2^) make the law ineffective and even discriminatory for most hospitality workers. The workers who were most exposed at baseline continue to be exposed after the law. These workers have higher levels of exposure to SHS, are exposed to tobacco-specific carcinogens, and more frequently have tobacco-related morbidity than workers in nonsmoking venues [39], [44], [45].

In terms of risk assessment, the *de minimis* risk for increased mortality is that level at or below which involuntary risk is generally of no regulatory concern (typically a 10^−6^ lifetime risk) and the *de manifestis* risk is that level at or above which involuntary hazards are invariably of regulatory concern (typically a 3.10^−4^ lifetime risk) [46]. The US Occupational, Safety & Health Administration (OSHA) considers a “significant risk” a 10^−3^ lifetime risk [47]. The *de manifestis* risk for SHS exposure occurs at salivary cotinine concentrations of 0.14 ng/ml [48]. An average salivary cotinine level of 0.4 ng/ml is associated with a probability of lung cancer death of 1 excess lung cancer death per 1000 workers (OSHA's “significant risk”) for a standard 45-year working lifetime, and with a probability of 1/100 for heart disease [47]. According to these estimates of lifetime risk, having a salivary cotinine concentration of 1.4 ng/ml increases the risk of lung cancer to 1/400 and the risk of heart disease to 1/30. Thus, median salivary cotinine post-ban concentrations in Spain were above the *de manifestis* acceptable levels of risk, regardless of the type of regulation of the venue.

The significant effects we observed in venues where smoking was completely prohibited in Spain are consistent with results from other countries with total bans. An early study in San Francisco (USA) showed improvements in respiratory health after the total ban, as well as a reduction in self-reported workplace exposure to SHS [8]. Similar findings, supported by significant reductions in biomarkers of individual exposure, were reported in studies from the USA [8], [9], [13], [14], Ireland [10], [11], [16], Scotland [15], [18], Norway [12], and Italy [17]. The reduction in exposure to SHS among workers at venues where smoking was completely banned was similar to that in previous studies regardless of the biomarker used (salivary cotinine [9]–[11], [16], [18], urinary cotinine [12], [13], [17], serum cotinine [15] or hair nicotine [14]).

Our data show that in venues where smoking is allowed in (at least in theory) physically separated areas, workers were not protected against SHS. These results are in agreement with previous studies in venues that had smoking rooms or implemented different levels of restriction. A cross-sectional study in New Zealand [49] found an inverse relationship between salivary cotinine concentrations in hospitality workers and the venues' smoking policy: the less restrictive the policy, the higher the cotinine concentrations (smoking in bars was not prohibited by law until December 2004). A similar pattern was observed in another cross-sectional study in Vancouver [50], with an almost 4-fold greater mean hair nicotine concentration in bar workers in venues where smoking was permitted, compared to workers in venues where smoking was completely prohibited. A study conducted in New York State to evaluate the impact of the Clean Indoor Air Act passed in 2003 showed no reductions in urinary cotinine concentrations among workers in American Indian-owned casinos exempt from the Act [13]. Furthermore, the lack of effect of the Spanish law among hospitality workers in venues with partial or no restrictions on smoking paralleled our findings in the control group of hospitality workers in Portugal and Andorra, where no anti-smoking legislation was in effect at the time of the study.

Some methodological aspects of our study deserve consideration. We were able to recruit a relatively large number of hospitality workers from different regions of Spain, and our follow-up rate (66.8%) was acceptable as compared to previous studies. There were no meaningful differences between participants who were successfully followed up and those who were lost to follow-up. When we assembled the cohort, we gave priority to adherence in order to avoid attrition. The lack of regional sampling frames precluded random geographical sampling, and we tried to avoid selection bias by using a nonproportional quota sampling approach. We tried to enhance internal validity by optimizing the quality of the measures and facilitating participation at follow-up.

Our study and the one by Allwright et al. [10], which evaluated the effect of the Irish smoking ban, are the only studies that included a control group. Because of the complexities of the interventions, control groups are not a must in research designed to evaluate the effects of public health interventions [51], [52]; in our study the nation-wide scope of the law precluded a control group within Spain. The data for a cohort of hospitality workers from Portugal and Andorra who were “not exposed” to a smoking ban allowed us to obtain a clearer understanding of the effects of the anti-smoking law in Spain.

One of the most original characteristics of this study is that we compared three groups of venues according to the type of anti-smoking regulation. Since we also used a comparison group, this design allowed us to contrast the efficacy of each level of regulation. However, this design also poses a potential limitation because of the relatively small numbers of workers in each group after stratification. The specific exceptions included in the law led us to stratify our sample into the three subgroups reported here. Our hypothesis when designing the study was that we would observe a reduction in cotinine concentrations in nonsmoker workers after the law. Equivalence tests are recommended for evaluative purposes in public health studies when no differences or disparities are to be tested [53], but larger sample sizes are needed [54]. The pre-post design, the paired statistical analysis, and the modelling helped to ensure the validity of our estimates, and most of the comparisons reported had an acceptable statistical power (>70%) for detecting statistical differences with a 5% alpha error.

It is notable that at baseline, salivary cotinine concentrations were already higher among workers in venues that chose not to implement no-smoking regulations after the law came into effect. Two complementary explanations might account for this finding. First, in venues whose proprietors changed to a smoke-free environment, a mechanism of voluntary adaptation might have been operating before the law came into effect. At the time of our pre-ban survey and sample collection (shortly before the law came into effect), better ventilation at venues already contemplating a complete ban on smoking might have resulted in better indoor air quality. Second, venues that did not implement a total or partial ban on smoking were mostly smaller than 100 m^2^ and had higher airborne nicotine concentrations at baseline [55], [56], a measure of air quality highly dependent on the total volume of the venue [57]. Moreover, we found, unexpectedly, that the prevalence of any respiratory symptom before the law in Spain, differed depending on the type of restriction after the law: prevalence was higher in venues that became totally smoke-free. However, these baseline differences, including the lower cotinine concentration in control participants in Portugal and Andorra, had no effect on before-after comparisons.

With regard to the methods we used for data collection, the validated questionnaire for SHS exposure [34], [35] and for respiratory health [37], [38], as well as the use of salivary cotinine concentration as a specific biomarker of SHS exposure in the previous 2–5 days [58], [59], are strengths of this study. Saliva samples were collected at different times of day during the workers' shifts, and hence systematic errors due to sampling time are unlikely. Moreover, we were able to adjust for day of the week in the multivariate models. The analytical method to evaluate salivary cotinine is highly sensitive, assessors of cotinine concentration were blind to the participants' smoking status, and the same protocol was used for all saliva samples [60].

We used a combination of a biomarker and self-reported exposure, as this is considered a good way to estimate exposure [61]. Self-reported exposure to SHS was recorded with a previously developed questionnaire for use in the general nonsmoking population, which has shown acceptable validity [30], [34]. Systematic error due to recall (wish or self-compliance bias) in the perception of exposure to SHS as well as in the reporting of respiratory symptoms cannot be disregarded [62], [63]. However, recall bias is unlikely since the decline in self-reported hours of exposure to SHS paralleled the decrease in salivary cotinine concentration.

In summary, the partial smoking ban in Spain does not sufficiently protect hospitality workers against SHS and its effects on respiratory health. These results provide further evidence in support of World Health Organization policy recommendations to protect workers and the population from exposure to SHS by means of total bans [19], [64]. Our findings suggest the need for significant changes in Spanish law to encourage total bans aimed at creating 100% smoke-free environments, with no exceptions [6], [65], [66]. In Spain, approximately 1 400 000 workers (7% of the working population) are employed in the hospitality sector [67]. It has been estimated that only 30% of all hospitality venues are actually smoke-free (premises smaller than 100 m^2^ which have gone smoke-free voluntarily, plus those larger than 100 m^2^). Hence, almost 1 000 000 hospitality workers (approximately half of them nonsmokers) in Spain continue to be unprotected against SHS. Policy makers in other countries currently considering the scope of their smoke-free legislation should not ignore these results. Partial bans, voluntary policies [68] or “courtesy of choice” programs (promoted by the tobacco industry) [69], [70] do not completely protect workers and others against second-hand smoke.

### The Spanish Smoking Law Evaluation Group

**Institut Català d**'**Oncologia:** Esteve Fernández (principal investigator)_,_ Marcela Fu, Jose M. Martínez-Sánchez, Anna Martín, Josep Maria Borràs, Stephanie Rania, Jorge Twose, Anna Schiaffino;

**Agència de Salut Pública de Barcelona:** Manel Nebot and Carles Ariza (principal investigators), María José López, Francesca Sánchez-Martínez, Francesc Centrich, Glòria Muñoz, Eulàlia Serrahima;

**Generalitat de Catalunya:** Esteve Saltó, Araceli Valverde, Meia Faixedas, Francesc Abella, Enric Rovira;

**IMIM-Hospital del Mar:** José Antonio Pascual, Raúl Pérez;

**Xunta de Galicia:** Mónica Pérez-Ríos (study coordinator), Begoña Alonso, María Isolina Santiago, María Jesús García, Míriam Otero;

**Govern de les Illes Balears:** Arturo López (study coordinator), Elena Tejera, Magdalena Borrás, Juan A. Ayensa, Ernesto Pérez;

**Generalitat Valenciana:** Francisco Carrión (study coordinator), Pepa Pont, José A. Lluch, Elena Pérez;

**Gobierno de Cantabria:** M. Eugenia López (study coordinator), Sonia Álvarez, M. Emma del Castillo, Fernando Martín, Blanca M. Benito;

**Junta de Extremadura:** José Antonio Riesco (study coordinator);

Comunidad de Madrid: Isabel Marta (study coordinator), Almudena García, Carmen Estrada, Virgilio Blanco;

**Gobierno de La Rioja:** Ana Esteban (study coordinator), M. Ángeles Hessel;

**Universidade do Minho:** José Precioso (study coordinator);

**Acadèmia de Ciències Mèdiques d**'**Andorra:** Margarida Coll (study coordinator).

## Supporting Information

Annex S1 (0.16 MB DOC)
