# Supplementary material for: Impact of the Spanish Smoking Law on Exposure to Second-Hand Smoke and Respiratory Health in Hospitality Workers: A Cohort Study
Source: PLoS One. 2009 Jan 23;4(1):e4244. doi: 10.1371/journal.pone.0004244 (PMC2621339; doi:10.1371/journal.pone.0004244)
Supplement: Annex S1 — (0.16 MB DOC) [file pone.0004244.s001.doc]

SPANISH VERSION

EXPOSICIÓN PASIVA AL HUMO AMBIENTAL DEL TABACO

**DOMICILIO**

**P47. ¿Algún miembro de su familia fuma habitualmente en su casa?**

| Sí |  | Especifique cuantas personas: |
| --- | --- | --- |
| No |  |  |
| No procede (vive solo/a) |  |  |
| NS/NC |  |  |

**P48. Durante la semana pasada, ¿cuántos cigarrillos se han fumado al día en su presencia en su casa?**

|  | Entre semana o día laborable | Fin de semana o tiempo libre |
| --- | --- | --- |
| Nº de cigarrillos |  |  |
| NS/NC |  |  |

# P49. ¿Es usted estudiante?

| Sí |  |  |  |
| --- | --- | --- | --- |
| No |  |  | Pasar a la pregunta 52 |
| NS/NC |  |  |  |

# P50. ¿En cuáles de estos espacios de tu facultad o centro de estudios hay señalización sobre consumo de tabaco? ¿Se cumple?

|  | ¿Se fuma? | | | Señalización | | |
| --- | --- | --- | --- | --- | --- | --- |
|  | Sí | No | NS/NC | Sí | No | NS/NC |
| Aula |  |  |  |  |  |  |
| Pasillo / Vestíbulo |  |  |  |  |  |  |
| Bar / Cafetería |  |  |  |  |  |  |
| Lavabo |  |  |  |  |  |  |
| Biblioteca |  |  |  |  |  |  |
| Sala de estudio |  |  |  |  |  |  |
| Copistería |  |  |  |  |  |  |

**P51. ¿Cuántas horas al día como promedio cree que está expuesto al humo ambiental del tabaco durante su estancia en la facultad o centro de estudios?**

| Nº de horas |  |
| --- | --- |
| NS/NC |  |

**TRANSPORTES**

P52. Durante la semana pasada, ¿ha ido en algún medio de transporte público?

| Sí |  |  | ¿Cuántas veces se ha fumado cerca de usted? | Nº veces |
| --- | --- | --- | --- | --- |
| No |  |  |  |  |
| NS/NC |  |  |  |  |

P53. Durante la semana pasada, ¿ha ido en algún medio de transporte privado?

| Sí |  |  | ¿Cuántas veces se ha fumado cerca de usted? | Nº veces |
| --- | --- | --- | --- | --- |
| No |  |  |  |  |
| NS/NC |  |  |  |  |

**TIEMPO LIBRE**

**P54. ¿Cuánto tiempo de «promedio» acostumbra a estar en ambientes con humo de tabaco fuera de casa y del trabajo?**

|  | Entre semana o día laborable | Fin de semana o tiempo libre |
| --- | --- | --- |
| Nada |  |  |
| Menos de 1 hora |  |  |
| De 1 a 4 horas |  |  |
| Más de 4 horas |  |  |
| NS/NC |  |  |

**LABORAL**

# P55. En su lugar de trabajo ¿hay algún tipo de regulación respecto al consumo de tabaco?

| Sí |  | ¿Desde cuándo? | Hace menos de un año  Hace un año o más  NS/NC |
| --- | --- | --- | --- |
| No |  |  | Pasar a la pregunta 57 |
| NS/NC |  |  | Pasar a la pregunta 57 |

# P56. ¿Las personas respetan esta regulación?

| Sí |  |
| --- | --- |
| No |  |
| NS/NC |  |

# P57. En su lugar de trabajo ¿existen espacios específicos para fumadores?

| Sí |  |
| --- | --- |
| No |  |
| NS/NC |  |

**P58. ¿Tiene usted algún compañero/a que fume cerca suyo en el trabajo?** (Que le llegue el olor del humo a su lugar de trabajo)

| Sí |  | Especificar cuántas personas: | |
| --- | --- | --- | --- |
| No |  |  | Pasar a la pregunta 60 |
| NS/NC |  |  | |

**P59. ¿Cuántas horas cree que está expuesto al humo ambiental del tabaco durante su jornada laboral?**

| Nº de horas |  |
| --- | --- |
| NS/NC |  |

ENGLISH VERSION

EXPOSURE TO ENVIRONMENTAL TOBACCO SMOKE

**AT HOME**

**P47. Does any member of your family usually smoke at home?**

| Yes |  | Specify how many persons: |
| --- | --- | --- |
| No |  |  |
| (Lives alone) |  |  |
| NS/NA |  |  |

**P48. In the last week, how many cigarettes has anyone smoked in your presence inside your home?**

|  | Working day | Non-working day |
| --- | --- | --- |
| Nº cigarettes |  |  |
| NS/NA |  |  |

# P49. Are you a student?

| Yes |  |  |  |
| --- | --- | --- | --- |
| No |  |  | Go to P 52 |
| NS/NA |  |  |  |

# P50. Which of these venues in your school/faculty/university has signs about smoking consumption?

|  | Smoking? | | | Signs | | |
| --- | --- | --- | --- | --- | --- | --- |
|  | Y | N | NS/NA | Y | N | NS/NA |
| Classroom |  |  |  |  |  |  |
| Corridors / hall |  |  |  |  |  |  |
| Bar / Cafeteria |  |  |  |  |  |  |
| Restrooms |  |  |  |  |  |  |
| Library |  |  |  |  |  |  |
| Study room |  |  |  |  |  |  |
| Photocopy shop |  |  |  |  |  |  |

**P51. How many ours per day do you think are you exposed to tobacco smoke at your school/faculty/university?**

| Nº hours |  |
| --- | --- |
| NS/NA |  |

**TRANSPORTS**

P52. During last week, have you used public transportation?

| Yes |  |  | How many times has anybody smoked in close proximity to you? | Nº times |
| --- | --- | --- | --- | --- |
| No |  |  |  |  |
| NS/NA |  |  |  |  |

P53. During last week, have you used private transportation?

| Yes |  |  | How many times has anybody smoked in close proximity to you? | Nº times |
| --- | --- | --- | --- | --- |
| No |  |  |  |  |
| NS/NA |  |  |  |  |

**LESIURE TIME**

**P54. How many hours per day have you spent in a place with tobacco smoke during your leisure not at home nor at work?**

|  | Working day | Non-working day |
| --- | --- | --- |
| None |  |  |
| Less than 1 hour |  |  |
| 1 to 4 hours |  |  |
| More than 4 hours |  |  |
| NS/NA |  |  |

**WORKPLACE**

# P55. Is there any type of rule about smoking in your work?

| Yes |  | Since? | Less than 1 year  1 year or more  NS/NA |
| --- | --- | --- | --- |
| No |  |  | Go to P 57 |
| NS/NC |  |  | Go to P 57 |

# P56. Does everybody respect this regulation?

| Yes |  |
| --- | --- |
| No |  |
| NS/NA |  |

# P57. Does your workplace have specific areas for smokers?

| Yes |  |
| --- | --- |
| No |  |
| NS/NC |  |

**P58. Does anybody smoke in close proximity to you at work?** (You can smell the tobacco smoke)

| Yes |  | Specify how many persons: | |
| --- | --- | --- | --- |
| No |  |  | Go to P 60 |
| NS/NA |  |  | |

**P59. How many hours per day do you think are you exposed to tobacco smoke at work?**

| Nº hours |  |
| --- | --- |
| NS/NA |  |
